# Allelic Frequency of Single-Nucleotide Polymorphisms in CYP2C9, VKORC1, and CYP4F2 Genetic Variants in Patients With Mechanical Heart Valves on Acenocoumarol Drug Therapy

**DOI:** 10.7759/cureus.115278

**Published:** 2026-08-27

**Authors:** Ahmad Najmi, Vikram Watti, Balakrishnan Sadasivam, Shubham Atal, Ashwin Kotnis, Jitendra Singh, Chenchula Santenna, Satyaprakash Vishwakarma, Pushpa Singh, Adila Zehra, Umra Baig

**Affiliations:** 1 Pharmacology, All India Institute of Medical Sciences, Bhopal, Bhopal, IND; 2 Cardiothoracic Surgery, All India Institute of Medical Sciences, Bhopal, Bhopal, IND; 3 Biochemistry, All India Institute of Medical Sciences, Bhopal, Bhopal, IND; 4 Translational Medicine, All India Institute of Medical Sciences, Bhopal, Bhopal, IND; 5 Biotechnology, Motilal Vigyan Mahavidyalaya, Barkatullah University, Bhopal, IND; 6 Biotechnology, Peoples University, Bhopal, IND

**Keywords:** acenocoumarol, anticoagulant, cyp2c9, cyp4f2, drug therapy, mechanical heart valve, pharmacogenetics, single nucleotide polymorphism, valvular heart disease, vkorc1

## Abstract

Background

Patients with mechanical heart valves require lifelong anticoagulation with vitamin K antagonists (VKAs), such as acenocoumarol. However, acenocoumarol has a narrow therapeutic index, and genetic variants in CYP2C9, VKORC1, and CYP4F2 significantly influence individual dose requirements and the risk of adverse events. Limited data exist on the prevalence of these clinically important variants in Central Indian populations.

Objectives

To determine the frequency of clinically significant genetic variants, alleles, and metabolizer phenotypes of the CYP2C9, VKORC1, and CYP4F2 genes, and to evaluate their clinical relevance in patients undergoing mechanical heart valve replacement with acenocoumarol anticoagulant therapy.

Methods

A total of 100 adults diagnosed with valvular heart disease with mechanical heart valve replacement and receiving acenocoumarol were enrolled from the cardiothoracic surgery department of All India Institute of Medical Sciences (AIIMS) Bhopal, India. Genotyping for CYP2C9*2, CYP2C9*3, VKORC1 c.-1639G>A, and CYP4F2 V433M variants was performed using TaqMan quantitative real-time polymerase chain reaction (qPCR). Ethical approval was obtained from the institutional human ethics committee, and written informed consent was obtained from all participants.

Results

Among the participants, 53% underwent mitral valve replacement, 31% underwent aortic valve replacement, and 16% underwent double-valve replacement. Rheumatic heart disease was the underlying cause in 95% of patients. Ischemic heart disease was the most common comorbidity. For CYP2C9*2, the major, minor, and heterozygous allele frequencies were 91%, 4%, and 5%, respectively. For CYP2C9*3, these were 86%, 9%, and 5%; for VKORC1, 68%, 3%, and 29%; and for CYP4F2, 60%, 8%, and 32%, respectively. The frequencies of normal, intermediate, poor, and rapid metabolizers were 36%, 44%, 15%, and 5%, respectively.

Conclusions

The study demonstrates a high prevalence (64%) of actionable CYP2C9, VKORC1, and CYP4F2 genotypes among the study participants. Genotype-guided dosing of acenocoumarol can potentially improve treatment outcomes and minimize adverse events in patients with valvular heart disease and mechanical valve replacement.

## Introduction

Patients with mechanical heart valve replacement need to take lifelong anticoagulation therapy. This is to prevent blood clots from forming on the valve [1,2]. Mechanical heart valves are more likely to get blood clots than tissue heart valves. Mechanical valves are more durable but require lifelong anticoagulation. Tissue valves do not require lifelong anticoagulation but wear out in 10-15 years. Thus, mechanical valves are preferred for younger patients, while tissue valves are better for older patients. The anticoagulant therapy helps reduce the risk of stroke, valve thrombosis, and other complications. Vitamin K antagonists (VKAs), such as warfarin and acenocoumarol, are widely used anticoagulants after heart valve replacement.

Traditional VKAs, such as warfarin and acenocoumarol, are the only FDA-approved oral anticoagulants for preventing valve thrombosis in mechanical heart valves. New oral anticoagulants (NOACs) or direct-acting oral anticoagulants (DOACs), such as dabigatran, apixaban, and rivaroxaban, are not FDA-approved for patients with mechanical heart valve replacements. In fact, they are strongly contraindicated for this purpose. Early clinical trials (e.g., the RE-ALIGN trial for dabigatran) showed that NOACs are associated with significantly higher rates of complications, including blood clots on the valve, strokes, and major bleeding, compared to older medications [3,4].

Acenocoumarol has a narrow therapeutic index; small changes in dosage can cause bleeding or thrombotic complications [5]. The acenocoumarol dosage can vary from person to person, depending on environmental factors and genetics. The main enzyme that metabolizes acenocoumarol in the liver is cytochrome P450 2C9 (CYP2C9), which is highly polymorphic. There are three main forms: CYP2C9*1 (normal or wild allele), CYP2C9*2, and CYP2C9*3. People with CYP2C9*2 and *3 variants have reduced enzyme activity, so they need lower doses of acenocoumarol.

However, there is limited data from Central India on how genetic variability affects acenocoumarol dosage. The Clinical Pharmacogenetics Implementation Consortium (CPIC) guidelines recommend genotype-based dosing for warfarin [6], but there is no specific recommendation for acenocoumarol. However, the Dutch Pharmacogenetics Working Group (DPWG) has recommended that patients with the VKORC1 (c.-1639G>A) AA genotype should be given 50% of the standard initial dose of acenocoumarol and undergo more frequent international normalized ratio (INR) monitoring. As per the DPWG, there are currently no recommendations for acenocoumarol dosing based on CYP2C9 genotypes [7]. This means that the patient's genetic profile should be considered for precision dosing of the VKA. It is essential to get the right dosage to prevent complications. An overdose of acenocoumarol can cause bleeding, and an underdose can cause blood clots. By considering a patient's genetic variability, more effective and safer anticoagulation therapy can be provided [8,9]. Although the FDA does not have official prescribing guidelines for acenocoumarol, the DPWG recommends that carriers of the VKORC1 genetic variants start at a much lower dose to prevent bleeding [10].

Therefore, the present study was conducted to determine the frequency of clinically significant genetic variants, alleles, and metabolizer phenotypes of the CYP2C9, VKORC1, and CYP4F2 genes and to evaluate their clinical relevance in patients undergoing mechanical heart valve replacement with acenocoumarol anticoagulant therapy.

## Materials and methods

Study population

The present prospective observational study was conducted at the Department of Pharmacology in collaboration with the Department of Cardiothoracic Surgery at All India Institute of Medical Sciences (AIIMS), Bhopal, a tertiary care teaching and referral hospital in Central India. A total of 100 adults diagnosed with valvular heart disease who had undergone mechanical heart valve replacement and were receiving acenocoumarol as maintenance anticoagulant therapy were enrolled from the Valvular Heart Disease and Anticoagulation Outpatient Department (OPD) of Cardiothoracic Surgery. The study was conducted over one year from May 2025 to April 2026.

Written informed consent was obtained from all participants prior to enrollment, and the study protocol was approved by the Institutional Human Ethics Committee (IHEC) of AIIMS Bhopal (approval no. IHEC-LOP/2025/IP028). The study was performed in accordance with the ethical principles outlined in the Declaration of Helsinki (1964) and its subsequent amendments.

Study inclusion criteria were adult patients of either gender with valvular heart disease who have undergone mechanical heart valve replacement and are taking acenocoumarol as an anticoagulant and are willing to provide written informed consent. The study exclusion criteria was patients who were taking other group of anticoagulants (direct thrombin inhibitors, factor X inhibitors, heparin etc), patients with history of severe liver disease or serum transaminase four times higher than normal, patients with history of severe renal impairment or serum creatinine > 2 mg/dL, and patients with congestive heart failure (ejection fraction < 40% or class IV symptoms) or concomitant intake of interacting drugs, such as amiodarone, rifampicin, phenytoin, carbamazepine, azoles, statins.

Genotyping

Genomic DNA was extracted from 5 mL of peripheral venous blood collected in ethylenediaminetetraacetic acid (EDTA)-containing Vacutainer tubes using the QIAamp® DNA Blood Mini Kit (Qiagen, Hilden, Germany) according to the manufacturer's protocol. DNA purity was assessed by measuring the absorbance ratio at 260 nm and 280 nm (A260/A280) using a NanoDrop™ 2000 spectrophotometer (Thermo Fisher Scientific, Waltham, MA). Samples with A260/A280 ratios outside the acceptable range of 1.8-2.0 were excluded from further analysis to ensure high-quality DNA for genotyping. DNA concentration was quantified and diluted to ≥20 ng/µL for downstream applications. All DNA samples were stored at -20°C until genotyping.

Genotyping was performed to detect the CYP2C9*2 (rs1799853), CYP2C9*3 (rs1057910), VKORC1 c.-1639G>A variants (rs9923231), and CYP4F2*3 variant (rs2108622), using the TaqMan assays (Thermo Fisher Scientific, Waltham, MA), with quantitative real-time polymerase chain reaction (qPCR) (Bio-Rad, Singapore). All assays were conducted in 96-well plates using a 10 µL reaction volume (5 µL Master mix, including 0.50 µL SNP Assay Mix, and 5 µL DNA containing ≥20 ng of genomic DNA). A positive control sample with known genotypes and a no-template control (NTC) were used in each run to monitor contamination.

Genotype-to-phenotype interpretation was conducted according to CPIC guidelines [6]. The activity score (AS) was used to determine the CYP2C9 phenotype from genotype. A normal-function allele was assigned a score of 1. The decreased function allele was given a score of 0.5, and no function allele was given a value of zero. Based on AS, the normal metabolizer (NM) phenotype was assigned a score of 2, the intermediate metabolizer (IM) phenotype was assigned a score of 1/1.5, and the poor metabolizer (PM) phenotype was assigned a score of 0/0.5. The rapid metabolizer (RM) phenotype was defined by the presence of the CYP4F2*3 (3/*3 or *1/*3) allele, which is associated with increased vitamin K metabolism and higher warfarin/acenocoumarol dose requirements, consistent with CPIC recommendations [6].

Data collection

Data collection was done with the help of an anonymized case record form, including demographic and socioeconomic details of the participants, diagnosis, type and date of surgery, clinical history, examination and investigation details, details of drug intake, INR chart, number of episodes with excessive INR, number of bleeding episodes, and the number of episodes with thromboembolism. Bleeding episodes were defined as major bleeding requiring hospitalization or transfusion. No major bleeding events occurred during the study period.

Statistical analysis

The continuous variables were expressed as mean ± standard deviation (SD) and compared using a t-test for independent samples. Categorical variables were expressed as percentages and compared using Pearson's chi-square test. SNP frequency, genotypes, allele status, and metabolizer phenotypes were assessed in patients undergoing mechanical heart valve replacement. Major, minor, and heterozygous allele frequencies were calculated and expressed as percentages. Weekly mean dose of acenocoumarol was compared among different phenotypes by using a one-way analysis of variance test (ANOVA), followed by Tukey's post hoc test. All statistical analyses were performed using Microsoft Excel (Microsoft® Corp., Redmond, WA) and Statistical Product and Service Solutions (SPSS, version 31.0.2.0; IBM SPSS Statistics for Windows, Armonk, NY). A total of 100 participants were recruited using convenience sampling for this cross-sectional analysis.

## Results

A total of 100 participants with valvular heart disease and mechanical heart valve replacement were recruited for the study from various districts of Madhya Pradesh and Chhattisgarh. Of the total, 53% (n = 53) underwent mitral valve replacement (MVR) for severe mitral stenosis or regurgitation, and 31% (n = 31) underwent aortic valve replacement (AVR) for severe aortic stenosis or regurgitation. Additionally, 16% (n = 16) of the remaining patients underwent double-valve replacement (mitral replacement and AVR). Rheumatic heart disease (RHD) was the most common cause of underlying valvular heart disease (95%, n = 95), followed by congenital (3%, n = 3) and degenerative (2%, n = 2). The median age was 43 years, with an interquartile range (IQR) of 33.0-54.3. Among the participants, 48% were male (n = 48), and 52% were female (n = 52). Median body mass index (BMI) among participants was 24.6, with an IQR of 22.1-27.3. The majority of participants belonged to the rural population (58%, n = 58), followed by the urban population (42%, n = 42). Ischemic heart disease was the most common comorbid condition, followed by diabetes mellitus (Table 1). 

**Table 1:** Demographic and clinical characteristics of the study population (N = 100) BMI: body mass index; IQR: interquartile range

Characteristic	Category	Value
Age (years)	Median (IQR)	43.0 (33.0-54.3)
BMI	Median (IQR)	24.6 (22.1-27.3)
Gender	Male	48 (48%)
	Female	52 (52%)
Residence	Rural	58 (58%)
	Urban	42 (42%)
Occupation	Business	35 (35%)
	Employed	29 (29%)
	Housewife	20 (20%)
	Farmer	11 (11%)
	Student	5 (5%)
Marital status	Married	56 (56%)
	Unmarried	44 (44%)
Diagnosis	Mitral valve replacement	53 (53%)
	Aortic valve replacement	31 (31%)
	Double valve replacement	16 (16%)
Etiology	Rheumatic heart disease	95 (95%)
	Congenital	3 (3%)
	Degenerative	2 (2%)
Comorbid illness	Ischaemic heart disease	15 (15%)
	Diabetes mellitus	7 (7%)
	Hypothyroidism	6 (6%)
	Deep vein thrombosis	3 (3%)

Acenocoumarol was the most commonly prescribed anticoagulant in 100% of participants (N = 100), and low-dose aspirin was used as the most commonly prescribed antiplatelet drug in 100% of participants (N = 100). Metoprolol extended release was prescribed in 41% of participants (n = 41). Fixed-dose combination of torasemide and spironolactone was prescribed in 39% of participants (n = 39). Penicillin G was prescribed for prophylaxis of rheumatic heart disease in 19% of participants (n = 19) (Table 2).

**Table 2:** Most commonly prescribed drugs FDC: fixed-dose combination; IU: international units

Drug Class	Drug	Daily dose	Number (Percentage)
Anticoagulant	Acenocoumarol	1-6 mg (as per INR)	100 (100%)
Antiplatelet	Low-dose aspirin	75-150 mg	100 (100%)
Beta blocker	Metoprolol (extended release)	25-100 mg	41 (41%)
Diuretic	FDC of torasemide and spironolactone	Torasemide 5-10 mg, spironolactone 25-50 mg	39 (39%)
Antibiotic	Penicillin G	400,000 IU	19 (19%)

For the CYP2C9*2 gene, the frequencies of the major, minor, and heterozygous alleles (C/A) were 91% (n = 91), 4% (n = 4), and 5% (n = 5), respectively. For the CYP2C9*3 gene, the frequencies of the major, minor, and heterozygous alleles were 86% (n = 86), 9% (n = 9), and 5% (n = 5), respectively. For the VKORC1 gene, the frequencies of major, minor, and heterozygous alleles were 68% (n = 68), 3% (n = 3), and 29% (n = 29), respectively. For the CYP4F2 gene, the frequencies of the major, minor, and heterozygous alleles were 60% (n = 60), 8% (n = 8), and 32% (n = 32), respectively (Table 3).

**Table 3:** Percentage distribution of major, minor, and heterozygous alleles G/G: wild normal variant genotype; A/A: homozygous mutant genotype; G/A: heterozygous mutant genotype

Gene	Major allele	Minor allele	Heterozygous
CYP2C9*2	91% (n=91)	4% (*2/*2) (n=4)	5% (*1/*2) (n=5)
CYP2C9*3	86% (n=86)	9% (*3/*3) (n=9)	5% (*1/*3) (n=5)
VKORC1	68% (G/G) (n=68)	3% (A/A) (n=3)	29% (G/A) (n=29)
CYP4F2*3	60% (n=60)	8% (*3/*3) (n=8)	32% (*1/*3) (n=32)

Mean ± SD dose (weekly maintenance dose in mg) of acenocoumarol among NM was 7.55 ± 2.23; mean ± SD dose (weekly maintenance dose) of acenocoumarol among IM was 4.50 ± 2.11; mean ± SD dose (weekly maintenance dose) of acenocoumarol among PM was 1.50 ± 1.11 (p value <0.001); and mean ± SD dose (weekly maintenance dose) of acenocoumarol among RM was 14.50 ± 4.99 (p value <0.0001) (Table 4).

**Table 4:** Weekly maintenance dose of acenocoumarol by metabolizer phenotype NM: normal metabolizer; IM: intermediate metabolizer; PM: poor metabolizer; RM: rapid metabolizer * Obtained using one-way ANOVA and Tukey's post hoc test

Phenotype	Mean dose (mg/week)	P value
Normal metabolizer (NM)	7.55 ± 2.23	Reference
Intermediate metabolizer (IM)	4.50 ± 2.11	0.002*
Poor metabolizer (PM)	1.50 ± 1.11	<0.001*
Rapid metabolizer (RM)	14.50 ± 4.99	<0.0001*

**Figure 1:**
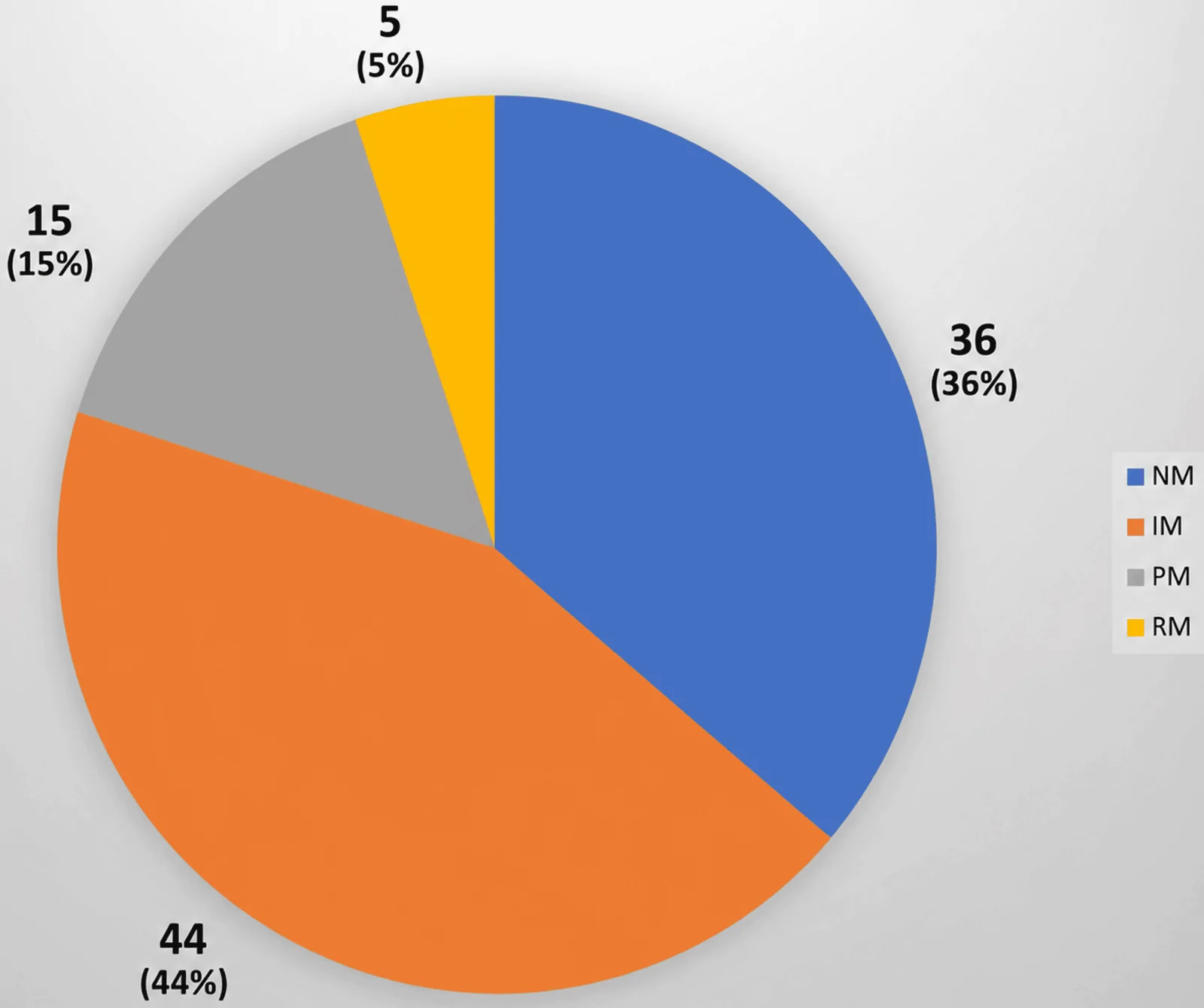
Percentage distribution of metabolizer phenotypes (%) NM: normal metabolizer; IM: intermediate metabolizer; PM: poor metabolizer; RM: rapid metabolizer

The percentage frequencies of NM, IM, PM, and RM were 36% (n = 36), 44% (n = 44), 15% (n = 15), and 5% (n = 5), respectively (Figure 1). 

**Figure 2:**
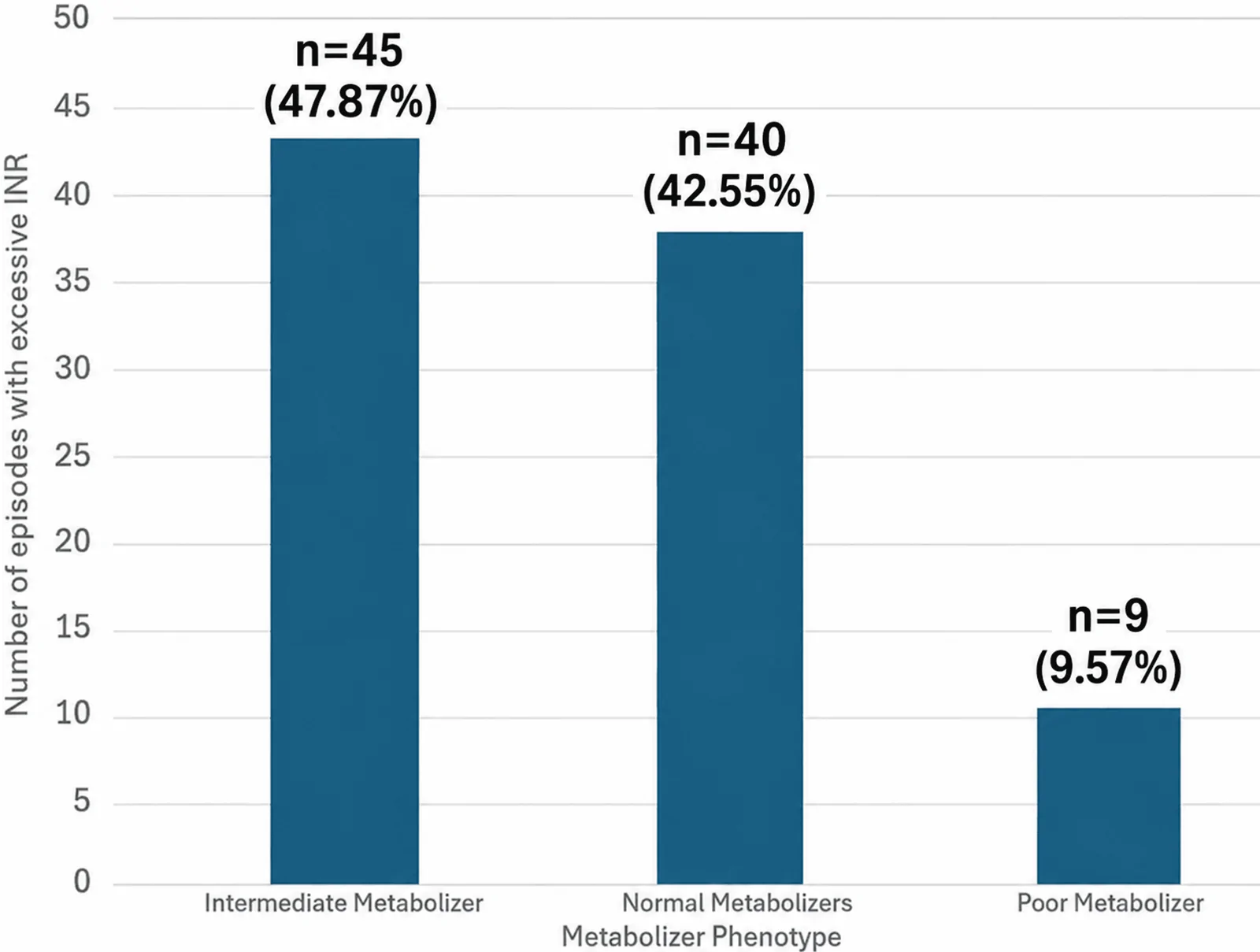
Number of episodes with INR >3 by metabolizer phenotype Values represent the total episodes recorded during the study period; some participants experienced multiple episodes.

In our study, the maximum episodes (47.87%) of raised INR (>3) were found among IM (n = 45) owing to their high frequency. This was followed by NM (42.55% or n = 40) and PM (9.57% or n = 9) (Figure 2). 

## Discussion

The study presents a comprehensive analysis of the frequency and clinical significance of CYP2C9, VKORC1, and CYP4F2 gene alleles, genotypes, and metabolizer phenotypes in a Central Indian population of 100 individuals diagnosed with valvular heart disease and mechanical heart valve replacement. Our findings contribute to scientific evidence indicating a high prevalence of actionable genotypes in the population of Central India. Both warfarin and acenocoumarol are VKAs; however, acenocoumarol is preferred over warfarin due to its salient pharmacokinetic properties. The half-life of acenocoumarol is much shorter (8-11 hours) than that of warfarin (30-60 hours). This means that both the onset and offset of acenocoumarol's action are faster, which is beneficial in patients with mechanical heart valves in case of dose adjustment. Due to its cost factor, acenocoumarol can be a better alternative to warfarin in India [11,12]. The risk of drug interaction is also less compared to warfarin due to less dependence on the CYP2C9 enzyme for drug metabolism. Some studies found that acenocoumarol provides better INR control than warfarin [12].

Comparative distribution of genotypes

In this study, the distribution of variant alleles of CYP2C9*2, CYP2C9*3, VKORC1, and CYP4F2*3 was analyzed in our population. Heterozygous CYP2C9 diplotype frequencies of *1/*2 and *1/*3 were 5% each in our study, while homozygous CYP2C9 *2/*2 and *3/*3 were 4% and 9%, respectively (Table 5). 

**Table 5:** Prevalence of SNPs in different populations ClinPGx = Clinical Pharmacogenetics Implementation Consortium reference population data [4] NA: not available/not reported in the original study

Population	*1/*2	*1/*3	*VKORC1 G/A	*VKORC1 A/A	*CYP4F2 *3/*3
Present study (Central India)	5%	5%	29%	3%	8%
North India (Choudhary et al.) [2]	7.3%	8.6%	29.3%	2.6%	NA
North India (Nahar et al.) [13]	9.6%	20%	34.4%	1.4%	NA
North India (Rathore et al.) [14]	9.3%	16.9%	29.3%	4%	NA
South India (Krishna Kumar et al.) [15]	6.7%	13.8%	18.8%	1.2%	NA
South India (Pavani et al.) [16]	4.25%	3.25%	11.9%	NA	NA
Caucasian (ClinPGx) [6]	20.17%	11.33%	41.24%	NA	NA
Middle Eastern [6]	20.32%	14.32%	46.52%	NA	NA
East Asian [6]	0.12%	6.5%	88.16%	NA	NA

VKORC1 heterozygous G/A and homozygous A/A mutant frequencies are 29% and 3%, respectively, which were approximately equal to previous published studies [2,13]. The genotype frequencies of CYP2C9 and the haplotype frequency of VKORC1-1639 are compared across several major Indian studies in Table 5. We observe that the *1/*2 genotype frequency of CYP2C9*2 and VKORC1-1639 are approximately equal to those reported in other North Indian population studies [2,13,14]. Compared with the South Indian population, the CYP2C9*3 mutant population is more prevalent in Central India [15,16]. In our study, a relatively higher proportion (9%) of mutant variants was observed for the CYP2C9*3 gene (Table 4). Compared with the global average frequencies of CYP2C9 diplotypes and VKORC1 G/A reported in the CPIC guideline [6], our population shows lower frequencies of the mutant diplotypes. Another salient feature of our study is that we genotyped CYP4F2*3, which was previously missing in previous studies. CYP4F2*3 genotyping is recommended by CPIC since this allele is associated with increased vitamin K metabolism and higher warfarin dose requirements [6].

The CYP4F2 gene encodes vitamin K oxidase, an enzyme responsible for metabolizing vitamin K. The CYP4F2*3 variant (V433M, rs2108622) reduces enzyme activity, leading to increased hepatic vitamin K levels. This mechanism counteracts the effects of VKORC1 variants by increasing the vitamin K available for carboxylation, thereby requiring higher VKA doses [16,17].

In our study, we observed a 32% heterozygous (*1/*3) and 8% homozygous mutant (*3/*3) frequency for CYP4F2*3. This represents a substantial proportion of patients (40%) who may require higher acenocoumarol doses than predicted by CYP2C9 and VKORC1 genotyping alone. Previous studies from North India did not include CYP4F2 genotyping, making our study the first to report these frequencies in Central India [2,11,12].

The CPIC guidelines recommend considering the CYP4F2*3 genotype for warfarin dosing, particularly when combined with CYP2C9 and VKORC1 genotypes [6]. Patients with CYP4F2*3 variants typically require 10-20% higher doses compared to wild-type carriers [6]. In our cohort, the presence of CYP4F2*3 in 40% of participants suggests that a significant proportion would benefit from genotype-guided dose adjustments to achieve therapeutic INR targets more rapidly and reduce the risk of over-anticoagulation.

Future studies should evaluate the combined effect of CYP2C9, VKORC1, and CYP4F2 genotypes on acenocoumarol dose requirements in Central Indian patients, as the interaction between these variants may be more complex than currently appreciated.

Distribution of metabolizer phenotypes

In our study, IM had the highest frequency (44% or n = 44), followed by normal metabolizers (36% or n = 36), poor metabolizers (15% or n = 15), and rapid metabolizers (5% or n = 5) (Figure 1). The study demonstrates a high prevalence (64% or n = 64) of actionable genotypes when combining IM, PM, and RM, underscoring the need for personalized acenocoumarol dosing. In a previous study conducted by Choudhary et al. [2], among the population of North India, the prevalence of NM and IM was 51.5% and 47.4%, respectively. This difference may be due to differences in the genetic profiles of patients from Central India. The participants in our study come from a diverse genetic background, including the tribal population. In our study, we found a significantly higher PM rate (15%) than in the previous study by Choudhary et al. [2].

Weekly maintenance dose of acenocoumarol in different metabolizer phenotypes

Mean ± SD dose (weekly maintenance dose) of acenocoumarol among NM was 7.55 ± 2.23 (Table 4). This dose was significantly higher among RM (p < 0.0001). The mean dose was significantly lower in IM (p < 0.002) and PM (p < 0.001). The US FDA has provided recommendations on initial warfarin dosing ranges for patients with different combinations of CYP2C9 and VKORC1 genotypes [17,18]. The difference in maintenance dose was statistically significant in intermediate, poor, and rapid metabolizers. This emphasizes the importance of genotype-guided dosing of acenocoumarol. Our results are in conformity with previously conducted studies [2]. 

Number of episodes with excessive INR

In our study, the maximum number of episodes of raised INR (>3) were found among intermediate metabolizers (n = 45 or 47.87%) owing to their high frequency (Figure 2). This was followed by normal metabolizers (n = 40 or 42.55%) and poor metabolizers (n = 9 or 9.57%). Since the poor metabolizer percentage was lower than that of intermediate and normal metabolizers, episodes of raised INR were fewer in our cohort. Some participants experienced multiple episodes. During the study period, no bleeding or thromboembolic episodes were recorded among study participants. There was a history of one bleeding episode (GI bleed) among one participant from the IM phenotype. According to the American College of Chest Physicians (ACCP) guideline, the target INR for mechanical heart valves typically ranges from 2.0 to 3.0 (target 2.5) [19].

Genotype-based algorithms and the Indian population

A number of validated algorithms, based on both genetic and clinical factors, have been published for estimation of stable VKA starting dosages [20,21]. The International Warfarin Pharmacogenetics Consortium (IWPC) and the Gage et al. algorithm [17] are two validated published algorithms for VKA dose estimation. These algorithms are generated from European and American-African datasets [22]. For acenocoumarol, Rathore et al. have developed a regression model for dose prediction [14]. This model showed a variation of 41.5% in the dose of acenocoumarol based on various genetic and clinical factors, such as age, gender, smoking, alcohol status, diet, etc. However, this regression model is specific to the North Indian population. The IWPC algorithm's flexibility to adapt to local populations is challenged by ethnicity, lifestyle factors, and the distribution of polymorphisms [23,24]. Various online warfarin dose calculators are also available that predict warfarin dose based on genetic and clinical factors, such as age, gender, height, smoking and alcohol status, and concomitant drugs [25].

Pharmacogenetic profiles and anticoagulant dosing recommendations as per the DPWG guidelines

DPWG has recommended that patients with VKORC1 (c.-1639G>A) AA genotype should be given 50% of the standard initial dose of acenocoumarol and undergo more frequent INR monitoring. As per DPWG, there are currently no recommendations for acenocoumarol dosing based on CYP2C9 genotypes [7]. 

Clinical implications

Frequent dose adjustments of anticoagulants to control INR are a major issue for valvular heart disease patients who underwent mechanical heart valve replacement [26,27]. When INR levels are too low, patients are more likely to have blood clots. On the other hand, when INR levels are too high, it can cause too much blood thinning, which increases the risk of major bleeding. To solve this problem, special algorithms can be used that take into account the patient's genetic information, such as their CYP2C9, VKORC1, and CYP4F2 genes [14,15]. These algorithms will help select the optimal VKA dose for each patient to keep their INR within a safe range. Based on the genetic profile, the dose can be adjusted more quickly, thereby reducing the risk of adverse events [28]. Only limited data are available from Central India on the prevalence of CYP2C9, VKORC1, and CYP4F2 gene polymorphisms in patients undergoing mechanical heart valve replacement. The participants in our study come from diverse genetic backgrounds, including scheduled tribes, such as the Gond and Bhil, which have a mix of Indo-European, Dravidian, and Austroasiatic ancestry [29,30]. Genotype-guided dosing or personalized dosing can improve treatment outcomes by minimizing adverse events and selection of optimum doses of anticoagulants. Limited availability of pharmacogenetic testing facilities and a lack of awareness are major challenges for the implementation of genotype-based dosing of anticoagulants in patients of mechanical heart valve replacement in India. 

Limitations

We have analyzed only clinically significant single-nucleotide polymorphisms (SNPs) involved in acenocoumarol metabolism. However, there are other SNP variants also, which are less common in the Indian population. We did not include other confounding factors, such as smoking status, alcohol intake, diet, and the role of the gut microbiome, in our study. We have considered only a hundred participants from a single tertiary care centre in Central India. Hence, we cannot generalize our results to the entire Indian population. In the future, more multicenter studies with large sample sizes and the inclusion of both genetic and clinical factors are required.

## Conclusions

This study demonstrates a high prevalence (64%) of actionable CYP2C9, VKORC1, and CYP4F2 genotypes among Central Indian patients with mechanical heart valves on acenocoumarol therapy. The predominance of IM (44%) and the substantial proportion of poor (15%) and rapid (5%) metabolizers underscores the critical need for personalized anticoagulation dosing in this population. During the study period, the highest number of excessive INR episodes occurred in IM, highlighting the real-world clinical impact of genetic variability on treatment outcomes.
